# Supplementary material for: The challenge of controlling an auditory BCI in the case of severe motor disability
Source: J Neuroeng Rehabil. 2024 Jan 18;21:9. doi: 10.1186/s12984-023-01289-3 (PMC10795353; doi:10.1186/s12984-023-01289-3)
Supplement: Supplementary file 1 — Additional file 1: Appendix. Computation of the classification posterior probability. [file 12984_2023_1289_MOESM1_ESM.docx]

Appendix: computation of the classification posterior probability

In each trial, we obtain four data features that constitute our full observation denoted by $\tilde{Y}=\left\{ Y_{SL},Y_{DL},Y_{SR},Y_{DR} \right\}$, where $Y_{SL}$ (respectively $Y_{DL}$, $Y_{SR}$ and $Y_{DR}$) indicates the features for the *standard_left* condition (respectively *deviant_left*, *standard_right* and *deviant_right*).

Applying Bayes rule, the posterior probability $q$ in favour of a “yes” answer writes:

| $q= p\left( "yes" \vert\tilde{Y} \right)=\frac{p\left( \tilde{Y} \vert"yes" \right).p\left( "yes" \right)}{p\left( \tilde{Y} \right)}$ | (A.1) |
| --- | --- |

Similarly,

| $p\left( "no" \vert\tilde{Y} \right)=\frac{p\left( \tilde{Y} \vert"no" \right).p\left( "no" \right)}{p\left( \tilde{Y} \right)}=1-q$ | (A.2) |
| --- | --- |

Assuming that prior to any observation, the “yes” and “no” answers are equiprobable, it follows that:

| $\log p\left( "yes" \vert\tilde{Y} \right)- \log p\left( "no" \vert\tilde{Y} \right)=\log p\left( \tilde{Y} \vert"yes" \right)- \log p\left( \tilde{Y} \vert"no" \right)$ | (A.3) |
| --- | --- |

Further assuming that the four data features are conditionally independent, meaning that knowing the nature of the BCI user response is sufficient to predict the observed data, it follows that:

| $p\left( \tilde{Y} \vert"yes" \right)=\prod_{k\in\left\{ SL,DL,SR,DR \right\}} p\left( Y_{k} \vert"yes" \right)$ | (A.4) |
| --- | --- |

Hence

| $\log p\left( \tilde{Y} \vert"yes" \right)=\sum_{k\in\left\{ SL,DL,SR,DR \right\}} p\left( Y_{k} \vert"yes" \right)$ | (A.5) |
| --- | --- |

And similarly,

| $\log p\left( \tilde{Y} \vert"no" \right)=\sum_{k\in\left\{ SL,DL,SR,DR \right\}} p\left( Y_{k} \vert"no" \right)$ | (A.6) |
| --- | --- |

Our approach is supervised. This means that for each of the four conditions, both $p\left( Y_{k} | "yes" \right)$ and $p\left( Y_{k} | "no" \right)$ are learnt from calibration data, for each participant. This means that for each new set of observation $\tilde{Y}$, one can readily compute the log-likelihood ratio $\log\frac{p\left( Y_{k} | "yes" \right)}{p\left( Y_{k} | "no" \right)}$ for each condition and hence the right-hand side term in the above equation (A.3), which we denote $\Delta$. It also expresses as follows:

| $\Delta=\sum_{k\in\left\{ SL,DL,SR,DR \right\}} \log\frac{p\left( Y_{k} \vert"yes" \right)}{p\left( Y_{k} \vert"no" \right)}$ | (A.7) |
| --- | --- |
| Since equation (A.3) also writes: |  |
| $\log\left( \frac{q}{1-q} \right)=\Delta$ | (A.8) |

This yields the following formula for the ensuing posterior probability of a “yes” response:

| $q=\frac{1}{1+e^{-\Delta}}$ | (A.9) |
| --- | --- |

Put simply, $q$ is the sigmoid transform of argument $\Delta$, which is the amount of empirical evidence in favour of answer “yes” compared to answer “no”.
